# Texture analysis of cardiovascular MRI native T1 mapping in patients with Duchenne muscular dystrophy

**DOI:** 10.1186/s13023-025-03662-y

**Published:** 2025-03-19

**Authors:** Mary Luz Mojica-Pisciotti, Tomáš Holeček, Věra Feitová, Lukáš Opatřil, Roman Panovský

**Affiliations:** 1 International Clinical Research Center, St. Anne’s University Hospital, Pekařská 53, 60200 Brno, Czech Republic; 2 International Clinical Research Center and Department of Medical Imaging, St. Anne’s University Hospital, Faculty of Medicine, Masaryk University, Pekařská 53, Brno, 602 00 Czech Republic; 3 Department of Biomedical Engineering, Brno University of Technology, Technická 3082, 60200 Brno, Czech Republic; 4 International Clinical Research Center and Department of Medical Imaging, St. Anne’s University Hospital, Pekařská 53, 60200 Brno, Czech Republic; 5 International Clinical Research Center and 1st Department of Internal Medicine/Cardioangiology, St. Anne’s University Hospital, and Faculty of Medicine, Masaryk University, Pekařská 53, 60200 Brno, Czech Republic

**Keywords:** Cardiac MRI, Texture analysis, Duchenne muscular dystrophy, Radiomics

## Abstract

**Background:**

Duchenne muscular dystrophy (DMD) patients are monitored periodically for cardiac involvement, including cardiac MRI with gadolinium-based contrast agents (GBCA). Texture analysis (TA) offers an alternative approach to assess late gadolinium enhancement (LGE) without relying on GBCA administration, impacting DMD patients’ care. The study aimed to evaluate the prognostic value of selected TA features in the LGE assessment of DMD patients.

**Results:**

We developed a pipeline to extract TA features of native T1 parametric mapping and evaluated their prognostic value in assessing LGE in DMD patients. For this evaluation, five independent TA features were selected using Boruta to identify relevant features based on their importance, least absolute shrinkage and selection operator (LASSO) to reduce the number of features, and hierarchical clustering to target multicollinearity and identify independent features. Afterward, logistic regression was used to determine the features with better discrimination ability. The independent feature inverse difference moment normalized (IDMN), which measures the pixel values homogeneity in the myocardium, achieved the highest accuracy in classifying LGE (0.857 (0.572–0.982)) and also was significantly associated with changes in the likelihood of LGE in a subgroup of patients with three yearly examinations (estimate: 23.35 (8.7), p-value = 0.008). Data are presented as mean (SD) or median (IQR) for normally and non-normally distributed continuous variables and numbers (percentages) for categorical ones. Variables were compared with the Welch t-test, Wilcoxon rank-sum, and Chi-square tests. A P-value < 0.05 was considered statistically significant.

**Conclusion:**

IDMN leverages the information native T1 parametric mapping provides, as it can detect changes in the pixel values of LGE images of DMD patients that may reflect myocardial alterations, serving as a supporting tool to reduce GBCA use in their cardiac MRI examinations.

**Supplementary Information:**

The online version contains supplementary material available at 10.1186/s13023-025-03662-y.

## Background

Duchenne muscular dystrophy (DMD) is a severe X-linked genetic disease that affects predominantly males (carrier females may also exhibit subtle myocardial changes), generating progressive muscle deterioration and severe motor impairments [1]. Considering their risk for developing cardiac pathologies [1], sufferers must be periodically examined for cardiac involvement, typically once yearly [2]. Their screening starts at a young age and might include cardiac magnetic resonance imaging (MRI) to characterize their myocardial tissue and evaluate and monitor their cardiac health [1–4]. This imaging modality encompasses advanced techniques, such as native T1 parametric mapping, which quantifies the longitudinal relaxation time and provides detailed information about tissue composition [5, 6], and late gadolinium enhancement (LGE), which assesses myocardial tissue and requires the use of gadolinium-based contrast agents (GBCA) [7, 8].

Even though LGE is highly reproducible, it may not be suitable for patients with renal impairment, as it has been shown that GBCA deposition, though without substantial clinical effects, may be associated with the development of nephrogenic systemic fibrosis (NSF) in this group [9–14]. Moreover, other effects of GBCA in different populations, such as gadolinium retention in the central nervous system [15], nonuniform deposition in the brain [9, 16], and mild and moderate adverse reactions have been reported [17]. Nonetheless, the adverse effects of GBCA use are an active research topic.

So far, no studies have focused on GBCA deposition specifically in DMD patients, although concerns about their general long-term safety persist [18, 19], including the need for a more conservative approach to GBCA use in those with documented LGE [20]. It has also been reported that DMD patients might be at risk of developing kidney impairment due to the nature of the disease [19], even in advanced stages [21], meaning that in such cases, GBCA administration may need to be avoided due to the risk of NSF and potential deposition effects. An additional but less frequently addressed concern, despite its clinical relevance, is the administration of GBCA in DMD patients with difficult intravenous access. Although different techniques are proposed in the literature to face this challenge in broader contexts [22], in DMD sufferers, it often requires multiple attempts and is not always successful, indicating that non-contrast cardiac MRI may be more suitable for these cases where GBCA administration is not possible or is contraindicated [4, 20]. Therefore, minimizing the need for contrast use while maintaining the diagnostic accuracy of cardiac MRI remains a critical challenge [20, 23]. DMD patients might benefit from alternative approaches, including computational-based ones, to fully take advantage of the diagnostic potential of native cardiac MRI methods.

A step towards reducing GBCA use in cardiac MRI in DMD patients is the application of texture analysis (TA), a radiomics-based technique focused on mining data contained in images to quantify features that allow the extraction of information about tissues’ underlying structure and possibly function [24–27]. Its application in MRI for several cardiac pathologies has increased lately [27–36], as it could promote a strategic shift toward a safer and more effective approach for yearly cardiac MRI controls. In DMD patients, TA could improve the capability of T1 parametric mapping to detect diffuse myocardial alterations, enhancing its diagnostic value. While alternative radiomics-based approaches exist [24, 25, 37], TA quantifies image heterogeneity, possibly correlating with fibrosis and microstructural changes [24, 25].

This study was necessary to explore the impact of TA in native T1 parametric mapping as a contrast-free, computational approach to assess LGE in DMD patients, addressing the need to reduce the use of GBCA in their monitoring, given the potential risks associated with repeated contrast exposure.

## Methods

We hypothesize that TA features extracted from cardiac MRI parametric native T1 maps can provide information about LGE in DMD patients, potentially reducing the need to use GBCA in their yearly controls. Therefore, this study aims to evaluate the prognostic value of selected TA features in the LGE assessment of DMD patients.

### Study population

We retrospectively included 67 patients diagnosed with DMD and analyzed the first cardiac MRI examination at the start of their regular screening. Fifteen were randomly selected and reserved for the first step of the study. The remaining patients with complete examinations, i.e., with LGE data (LGE + and LGE-), were matched by age using optimal full matching with a logistic regression model to estimate the probability of LGE status [38, 39]. This approach creates subclasses that minimize age differences, resulting in balanced groups for comparison. Exams without LGE images corresponded to examinations where inserting the intravenous contrast bolus was not possible because of difficult venous access.

The cardiac MRI examinations were performed from December 1st, 2016, to December 2nd, 2022. These patients were earlier screened by the Parent Project from the Czech Republic [40–43] following the principles from the Declaration of Helsinki (2000) by the World Medical Association. The institutional ethics committee at the Hospital approved the original study (reference number blinded for review). All participants had written informed consent from their legally authorized representatives or the subjects themselves. Their clinical data was retrieved from the Hospital Information System.

### Cardiac MRI data acquisition

Cardiac MRI studies were performed on a 1.5 T scanner (Ingenia, Philips Medical Systems) following a standard protocol [44]. Long-axis and short-axis cine images were acquired with balanced turbo field echo steady-state free precession (SSFP) sequences. T1 parametric mapping was performed at the mid-ventricular level in the short-axis orientation using a modified Look-Locker inversion recovery sequence (MOLLI) before and 15 min after a contrast bolus injection [0.2 mmol/kg, gadobutrol (Gadovist, Bayer)]. MOLLI acquisition schemas of 5(3)3 and 4(1)3(1)2 were used for native and post-contrast T1 maps, respectively.

Finally, LGE images were acquired approximately 10 min after the contrast bolus injection with an inversion-recovery turbo field echo (IR-TFE) sequence, revealing contrast-enhanced areas in both long- and short-axis views. Typical parameters for selected cardiac MRI sequences in the protocol are listed in Table 1.

**Table 1:** Cardiac MR imaging protocol typical parameters

Parameter	Cine SSFP	MOLLI	LGE
Field of view (mm)	300 × 300	300 × 300	319 × 319
Acquisition voxel size (mm)	1.67 × 1.67 × 8.00	2.00 × 2.00 × 10.00	1.60 × 1.75 × 10.00
Reconstruction matrix	256 × 256	256 × 256	288 × 288
SENSE factor	1.5	2.0	2.5
Cardiac phases per RR interval	30 to 50	-	-
TE/TR	1.71/3.4	0.91/2.0	1.24/4.0
Bandwidth (Hz)	1102.3	1082.0	304.9
Flip angle	60°	35°	15°

Abbreviations: late gadolinium enhancement, LGE; modified Look-Locker inversion recovery sequence, MOLLI; sensitivity encoding, SENSE; steady-state free precession, SSFP; echo time, TE; repetition time, TR

### Clinical assessment

An expert radiologist performed the clinical assessment according to the established clinical protocols [45] using the IntelliSpace Portal (ISP) workspace (version 11, Philips Healthcare). Quantitative assessment of the left ventricular (LV) and right ventricular (RV) functions was described through the ejection fraction (EF), end-diastolic volume (EDV), end-systolic volume (ESV), stroke volume (SV), and cardiac output (CO). The assessment used cine images and also included the LV mass (LVM), tricuspid annular plane systolic excursion (TAPSE), mitral annular plane systolic excursion (MAPSE), and RV wall thickness (RVWT). LV and RV volumes were indexed to the body surface area (BSA), indicated at the end of the abbreviations with the letter I. Two clinical experts assessed LGE by identifying an area of contrast enhancement where the signal was higher than the mean signal intensity of a reference myocardium zone.

### Cardiac MRI T1 mapping assessment

Using the commercial software cvi42 (release 5.13.9, Circle Cardiovascular Imaging, Calgary, Canada), two experienced readers performed T1 parametric mapping assessments. The readers were blinded to the personal and clinical data. They traced the endocardial and epicardial contours in a mid-ventricular slice, excluding the trabeculae and the myocardial blood pool. Then, they verified the alignment in each slice and registered the images with a contour-based registration method. Finally, they generated and exported the motion-corrected native T1 maps for further analysis.

### Texture analysis

A successful pipeline was developed to perform the texture analysis in all patients using PyRadiomics (v3.1.0) [46] and Python (v3.7.16). DICOM images of motion-corrected native T1 maps were converted to NIfTI (Neuroimaging Informatics Technology Initiative) format for further processing. The corresponding myocardial mask, i.e., the endocardial and epicardial contours, was obtained with an in-house algorithm that extracted contour coordinates and mapped them onto the native T1 map image. The images were resampled to 1.17 × 1.17 mm to ensure consistency.

TA was performed on the original images with intensities normalized between µ ± 3σ (µ, the mean value of the gray levels inside the myocardium, and σ, the standard deviation) [47] without and with different filters: gradient filter, square filter, local binary pattern filters with 8 points and radius 1 and 2 (LBP(8,1) and LBP(8,2), respectively), and 2D discrete wavelet low- and high-frequency sub-bands filters (LL, LH, HL, HH).

### Texture feature selection

Feature selection started by identifying highly reproducible features by the inter-rater reliability through the intraclass correlation coefficient (ICC) and the coefficient of variation (CV). Two readers independently analyzed 15 randomly selected studies, and one repeated the analysis one month later. These studies were removed from the primary dataset to minimize potential data dependencies. Features with both intra- and interobserver ICC ≥ 0.75 and CV ≤ 10% were selected for further analysis [29, 48].

The dataset was randomly split into training (67%) and testing (33%) cohorts, and the classes were balanced to ensure the same number of LGE + and LGE- cases. To further reduce the dimension, two selection algorithms were independently applied to the highly reproducible features data from the training cohort: Boruta, which performs a top-down search to identify relevant features according to their importance [49], and least absolute shrinkage and selection operator (LASSO) with fivefold cross-validation, which reduces features by penalizing the coefficients of the less important ones [50]. The predictors were scaled before running the Boruta selection algorithm to minimize the risk of overfitting, ensuring that all features contributed equally to the model. Using both Boruta and LASSO as complementary algorithms helped ensure that the set of features was consistently identified across different approaches, enhancing the robustness of the feature selection process. Afterward, hierarchical clustering was applied to all variables selected by Boruta and LASSO target multicollinearity and identify independent features, which were compared between the LGE subgroups.

### Texture feature analysis

Multiple logistic regression models were trained with each independent feature to identify the features with better discrimination ability. 10-fold cross-validation was performed within each model. The Akaike Information Criterion (AIC) was used to select the best model. All the features were z-score normalized. A decision tree model based on the identified features was applied to determine a set of rules to discriminate for the presence of LGE.

Once significant features were identified, those were extracted for a subgroup of 15 DMD patients from the original cohort with at least three consecutive yearly examinations to assess their influence on the likelihood of having LGE. These were chosen to coincide with changes in LGE status if the patient exhibited it and to minimize bias. A general linear mixed-effects (GLM) model fitted by maximum likelihood (Laplace approximation) was used to study the influence of these features on the presence of LGE by calculating the log odds of LGE occurrence based on the identified features from the first-year native T1 maps.

### Statistical analysis

Descriptive statistics are reported as the mean (standard deviation, SD) or median (interquartile range, IQR) for normally and non-normally distributed continuous variables, respectively, and as numbers (percentages) for categorical ones. The normality of the data was checked by the Shapiro-Wilk test and visual inspection of the histograms. Proportions of categorical variables were analyzed using the Chi-square test of independence. The Welch two-sample t-test and Wilcoxon rank-sum test were used to compare normally and non-normally distributed variables. The adjusted P-value was obtained by using a false discovery rate correction. A P-value < 0.05 was considered statistically significant. The sample size was determined conservatively assuming Cohen’s d = 0.9, a large but realistic effect size in clinical studies, to achieve a power of 0.8 with a significance level of 0.05. Correlations were calculated using Pearson or Spearman for normally and non-normally distributed variables. The intraobserver and interobserver agreement was assessed with the ICC (type C, two-way mixed-effects model) was determined from fifteen randomly selected cases analyzed by two readers, one of whom repeated them one month apart. The repeatability was classified as poor (< 0.5), fair (0.50 to 0.75), good (0.75 to 0.90), and excellent (0.90 to 1) [51]. The CV was determined as the standard deviation and mean ratio. The Variance Inflation Factor (VIF) was determined to assess the multicollinearity of the final independent selected features.

Logistic regression models with 10-fold cross-validation were fitted and compared using the Akaike Information Criterion (AIC) to estimate the probability of a binary outcome. Cohen’s kappa was determined to quantify the level of agreement between observed and predicted classifications. Receiver operating characteristic (ROC) analyses were performed to assess the performance of the classification models. The area under the curve (AUC) was reported along with 95% confidence intervals (CI) to quantify the uncertainty of the estimates. Accuracy, balanced accuracy, and F1 score were also determined to evaluate each model’s predictive ability.

Longitudinal data analysis was conducted to examine changes in the independent feature with the highest classification accuracy over time for each patient with a generalized linear mixed (GLM) model fitted with maximum likelihood estimation with Laplace approximation. The GLM assessed the influence of the feature on the log odds of LGE presence. The model’s fit was evaluated using AIC and Bayesian Information Criterion (BIC). All statistical analyses were performed with R-4.3.0 and RStudio IDE (2023.03.0 + 386, RStudio, PBC), using base R [52], Boruta (v8.0.0, for Boruta selection) [49], brglm (v0.7.2, for fitting generalized linear models with bias-reduced estimators) [53], caret (v6.0-94, for machine learning algorithms and tools) [54], glmnet (v4.1-8, for fitting generalized linear models with elastic net regularization) [55, 56], ICC (v2.4.0, for calculating intraclass correlation coefficients) [57], MatchIt (v4.5.5, for propensity score matching) [38], and pROC (v1.18.5, for analyzing ROC curves and calculating AUC) [58].

## Results

### Study group

**Fig. 1:**
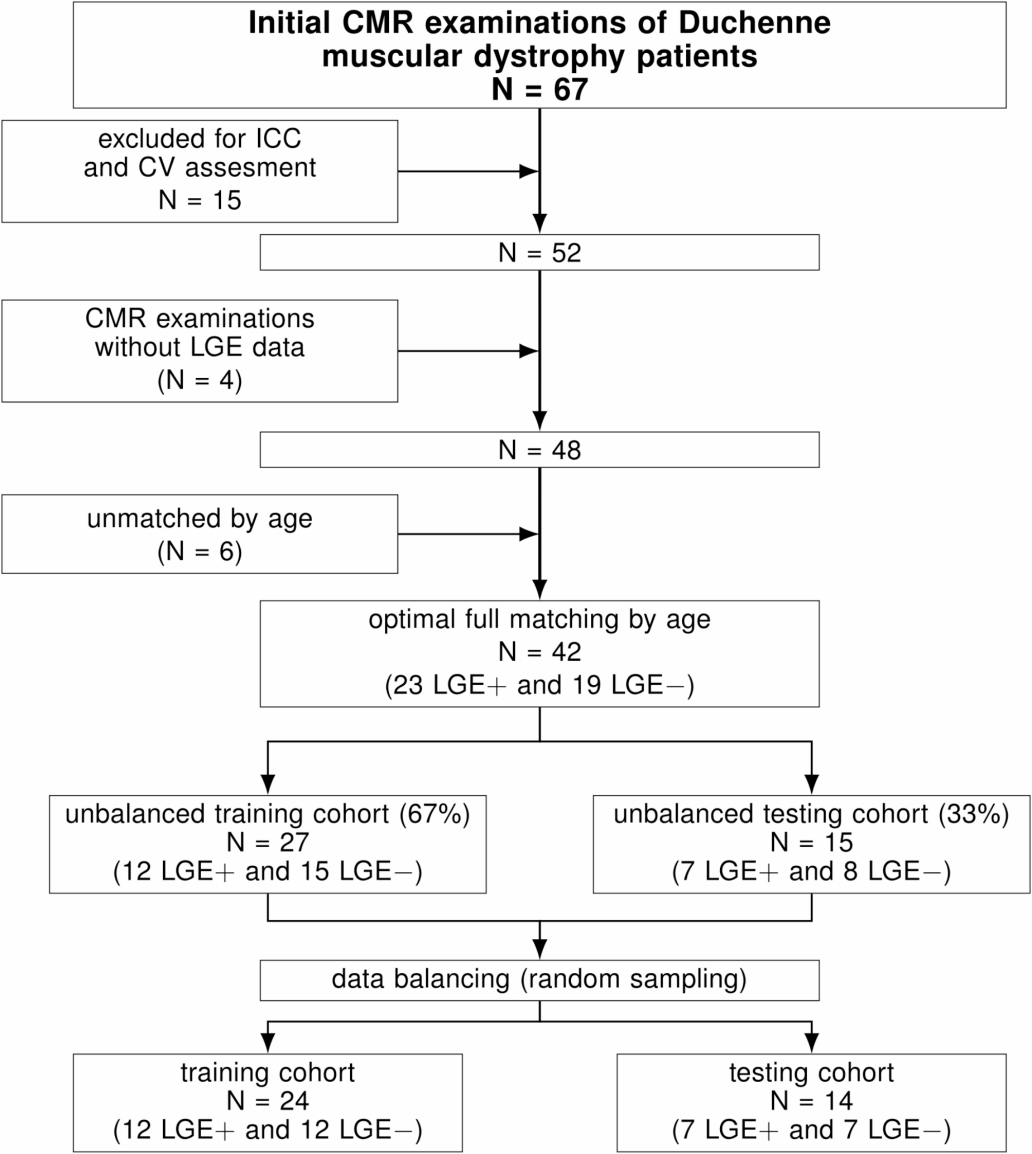
Study flowchart

Fifteen cardiac MRI studies were used for identifying highly reproducible features, and 52 for the primary analysis. Forty-eight examinations had LGE assessment, the remaining four corresponded to examinations performed on DMD patients with difficult peripheral venous access. Patients with and without LGE (LGE + and LGE-, respectively) were matched by age, and 42 remained for further analyses, 23 LGE + and 19 LGE-. The LGE patterns were intramural (12 patients), subepicardial (7 patients), and transmural (4 patients). The study flowchart is shown in Fig. 1. The participant characteristics are listed in Table 2. Patients without LGE had significantly higher LVEF than those with LGE, although it was preserved in both groups.

**Table 2:** General characteristics and left and right ventricular function between DMD patients according to LGE

Variable	LGE-, *N* = 19	LGE+, *N* = 23	*p*-value
Age (y)	12.6 (3.3)	13.8 (3.4)	0.256
BMI (kg/m^2^)	21.0 (5.2)	23.6 (6.0)	0.138
BSA (m^2^)	1.24 (1.04, 1.45)	1.31 (1.16, 1.63)	0.220
HR (bpm)	95 (11)	100 (15)	0.241
LVEF (%)	69 (64, 76)	58 (51, 66)	**0.001**
LVEDVI (ml/m^2^)	55.3 (41.0, 58.2)	52.6 (46.2, 62.7)	0.468
LVESVI (ml/m^2^)	15.2 (12.5, 20.5)	20.4 (16.2, 28.5)	**0.014**
LVSVI (ml/m^2^)	35.7 (8.3)	32.0 (7.0)	0.130
LVMI (g/m^2^)	36.6 (30.8, 38.4)	42.8 (35.8, 47.2)	**0.015**
MAPSE IVS (mm)	11.21 (1.78)	10.07 (1.51)	**0.033**
MAPSE FW (mm)	11.11 (1.73)	10.41 (2.10)	0.249
MAPSE (average) (mm)	11.16 (1.47)	10.24 (1.51)	0.054
RVEF (%)	67 (64, 74)	63 (52, 70)	0.065
RVEDVI (ml/m^2^)	53.8 (12.2)	47.5 (11.0)	0.091
RVESVI (ml/m^2^)	17.4 (14.2, 19.7)	17.4 (11.7, 21.4)	0.841
RVSVI (ml/m^2^)	34.1 (10.5)	30.0 (7.3)	0.156
TAPSE (mm)	18.0 (3.5)	17.8 (3.3)	0.823
RVWT (mm)	2.00 (2.00, 2.00)	2.00 (2.00, 2.12)	0.124

Variables are expressed as mean (standard deviation) or median (interquartile range) for normally distributed and non-normally distributed continuous variables. Abbreviations: BMI, body mass index; BSA, body surface area; DMD, Duchenne muscular dystrophy; LV, left ventricle; EF, ejection fraction; EDV, end-diastole volume; ESV, end-systole volume; FW, free wall; HR, heart rate; I, indexed; IVS, interventricular septum; LGE, late gadolinium enhancement; LVM, left ventricular mass; MAPSE, mitral annular plane systolic excursion; RV, right ventricle; SV, stroke volume; TAPSE, tricuspid annular plane systolic excursion; WT, wall thickness

### Cardiac MRI T1 mapping

**Fig. 2:**
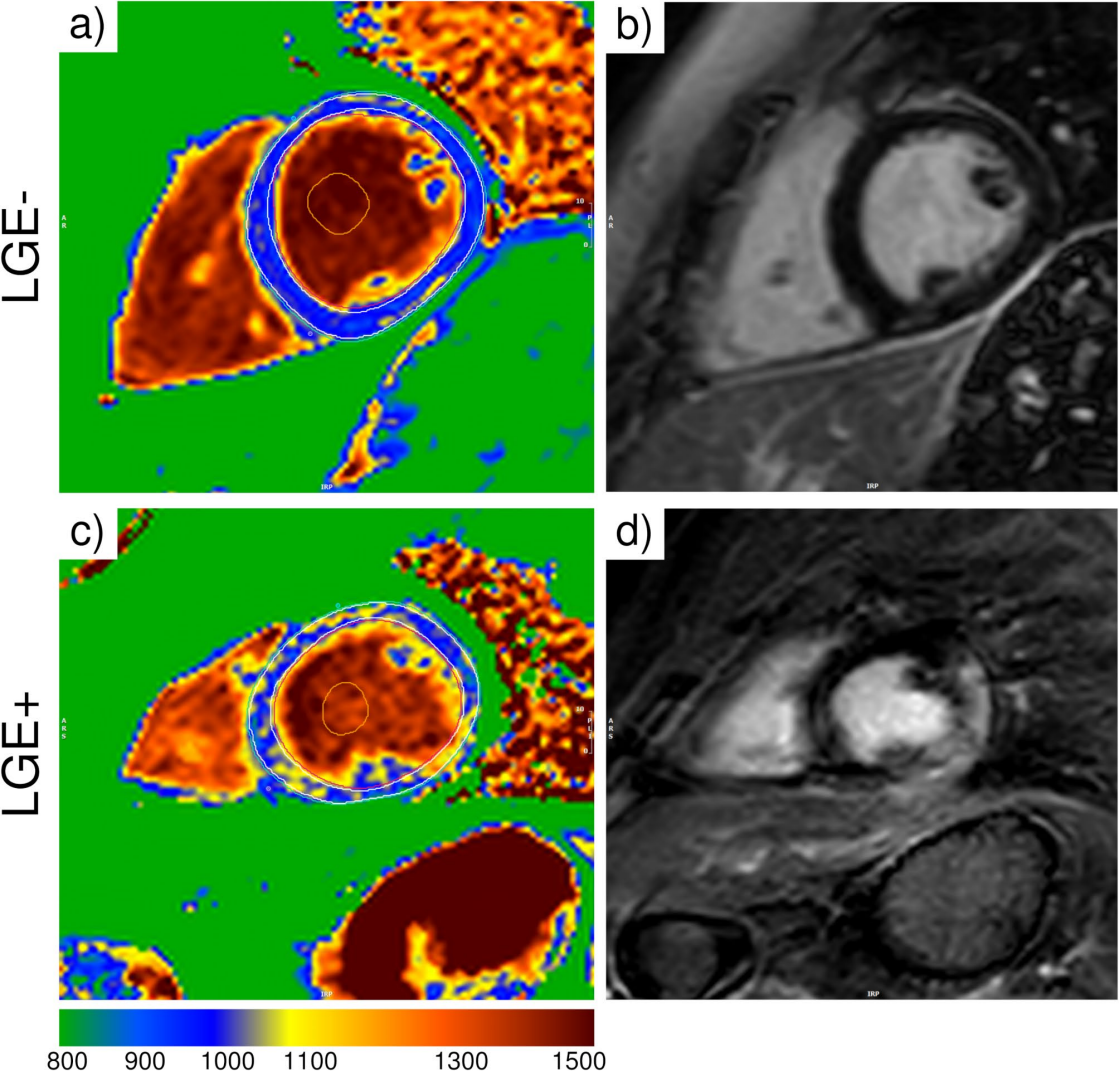
Representative native T1 maps and late gadolinium enhancement (LGE) images. Panels: **a**) native T1 map for the LGE- patient; **b**) LGE image for the LGE- patient; **c**) native T1 map for the LGE + patient; **d**) LGE image for the LGE + patient

The cardiac MRI native T1 mapping analysis is shown in Table 3. The native T1 longitudinal relaxation time significantly differed for the anterior segment between DMD patients according to their LGE status. An example of native T1 maps and LGE images for two patients is shown in Fig. 2.

**Table 3:** Cardiac MRI native T1 mapping for DMD patients according to LGE

Variable	LGE-, *N* = 19	LGE+, *N* = 23	*p*-value
T1 native (ms)			
S1 - Anterior	1016 (1006, 1035)	1053 (1019, 1068)	**0.035**
S2 - Anteroseptal	1020 (1004, 1041)	1027 (1016, 1052)	0.280
S3 - Inferoseptal	1022 (33)	1037 (35)	0.186
S4 - Inferior	1022 (992, 1045)	1035 (998, 1059)	0.200
S5 - Inferolateral	1032 (48)	1056 (78)	0.218
S6 - Anterolateral	1022 (41)	1042 (53)	0.195
Global	1032 (1002, 1041)	1044 (1013, 1059)	0.065

Variables are expressed as mean (standard deviation) or median (interquartile range) for normally and non-normally distributed continuous. Abbreviations: DMD, Duchenne muscular dystrophy; LGE, late gadolinium enhancement; S, segment

### Texture analysis

We obtained 918 features per case, including 9 shape-related features, 18 first order, 24 Gy-level co-occurrence matrix (GLCM), 14 Gy-level dependence matrix (GLDM), 16 Gy-level run length matrix (GLRLM), 16 Gy-level size-zone matrix (GLSZM), and 5 neighbouring gray-tone-difference matrix (NGTDM). The description and feature names are shown in Supplementary Table S1. The analysis took 11.68 ± 7.22 s per patient in an Intel(R) Core(TM) i7-8550U CPU @ 1.80 GHz. The results of all texture features were exported for further processing.

### Texture feature selection

Of the 918 features, 62 were highly reproducible ones. The group of 42 patients (23 LGE + and 19 LGE-) was randomly split into training (15 LGE + and 12 LGE-) and testing (8 LGE + and 7 LGE-). After balancing the classes, 24 patients remained in the training and 14 in the testing cohorts. The Boruta algorithm selected 20 features using the training cohort; the corresponding importance plot is shown in Supplementary Fig. 1. The Lasso algorithm selected 11 features; the relative feature importance is shown in Supplementary Fig. 2. Three of the identified features were common to both methods, as each selection method focuses on different data patterns: Boruta captures overall importance, while Lasso emphasizes individual associations with the outcome.

Afterward, five features were identified as independent after a hierarchical clustering algorithm was applied: two first-order (root mean squared (RMS)) with filter LBP (8,1) and entropy), two gray-level co-occurrence matrix (inverse difference moment normalized (IDMN) with filter wavelet LL and sum entropy with filter gradient), and one gray-level size-zone matrix (zone%) with filter LBP (8,1). Specifically, RMS is the square root of the mean of all the squared pixel values and measures the magnitude of the image, entropy quantifies the unpredictability of the image’s pixel values, IDMN measures the homogeneity of the pixel values, sum entropy quantifies the cumulative variation of pixel intensities across neighbouring pixels, and zone% measures the roughness or coarseness of the texture by zones in the region of interest [46]. The Variance Inflation Factor (VIF) was less than 5 in all cases (see Supplementary Table S2).

**Fig. 3:**
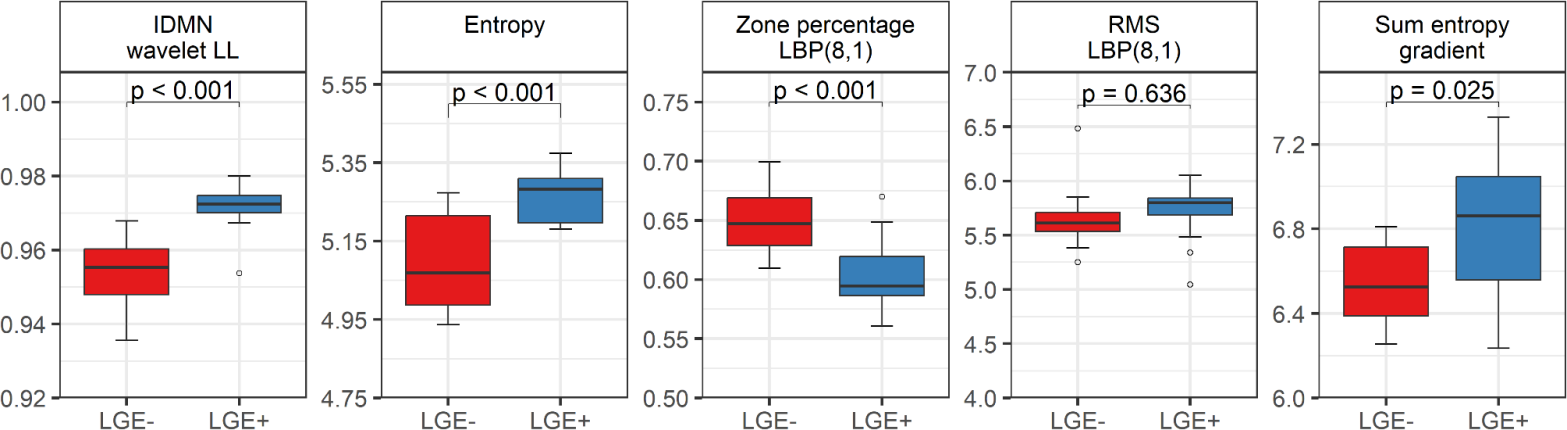
Distribution of selected feature values for DMD patients with and without LGE. Abbreviations: IDMN, inverse difference moment normalized; LBP, local binary pattern; LL, low-low; RMS, root mean squared

Finally, four selected features significantly differed for DMD patients with and without LGE: IDMN, entropy, zone%, and sum entropy. Their comparison is shown in Table 4; Fig. 3.

**Table 4:** Comparison of texture features in DMD patients according to LGE

Variables	Type	Filter	LGE-, *N* = 12	LGE+, *N* = 12	*p*-value
IDMN	GLCM	wavelet LL	0.954 (0.009)	0.972 (0.007)	**< 0.001**
Entropy	First order	-	5.07 (4.98, 5.22)	5.28 (5.20, 5.31)	**< 0.001**
Zone%	GLSZM	LBP(8,1)	0.65 (0.03)	0.60 (0.03)	**< 0.001**
RMS	First order	LBP(8,1)	5.66 (0.31)	5.72 (0.29)	0.636
Sum entropy	GLCM	gradient	6.53 (0.20)	6.80 (0.34)	**0.025**

Variables are expressed as mean (standard deviation) or median (interquartile range) for normally and non-normally distributed continuous. Abbreviations: DMD, Duchenne muscular dystrophy; GLCM, gray-level co-occurrence matrix; GLSZM, gray-level size-zone matrix; IDMN, inverse difference moment normalized; LBP, local binary pattern; LL, low-low; RMS, root mean squared

### Texture feature analysis

The logistic regression model with the IDMN feature achieved the highest accuracy in classifying the samples (accuracy 0.857 (0.572–0.982)) compared to the other significant features, as shown in Table 5. The area under the ROC curve (AUC) for the model with IDMN was 0.889 (95% CI: 0.721, 0.996), indicating good discriminative ability. Additionally, when adjusting models with up to two features, no improvement in accuracy or other metrics was observed. Also, for such a small size, using models with more than two features compromises statistical reliability and overfitting, making using single features a more viable approach for this analysis.

**Table 5:** Performance metrics for models with significant features identified by TA and global native T1

Model	Accuracy (95% CI)	AUC (95% CI)	Balanced accuracy	Kappa	F1 Score	AIC
IDMN	0.857 (0.572, 0.982)	0.889 (0.721, 0.996)	0.857	0.714	0.875	18.6
Entropy	0.786 (0.492, 0.953)	0.792 (0.583, 0.995)	0.786	0.571	0.800	22.6
Zone%	0.643 (0.351, 0.872)	0.675 (0.425, 0.896)	0.643	0.286	0.706	26.6
Sum entropy	0.643 (0.351, 0.872)	0.646 (0.396, 0.871)	0.643	0.286	0.667	31.6
Native T1 global	0.571 (0.289, 0.823)	0.578 (0.313, 0.830)	0.571	0.143	0.625	34.3

Variables are expressed as mean (standard deviation) or median (interquartile range) for normally and non-normally distributed continuous. Abbreviations: CI, confidence interval; IDMN, inverse difference moment normalized

A decision tree classifier was trained, and an optimal threshold of 0.969 for the IDMN feature was determined for classifying the presence of LGE. Such a choice achieved good discriminative ability (accuracy 0.857 (0.571, 0.982)). The AUC was 0.887 (95% CI: 0.643, 0.995), indicating strong LGE classification performance.

Afterward, the IDMN feature was extracted for a subgroup of 15 DMD patients with at least three consecutive yearly examinations for a preliminary evaluation. A significant effect of IDMN on the log odds of LGE was found (estimate: 23.35 (8.7), p-value = 0.008). The GLM showed a good fit to the data (AIC 33.8, BIC 42.6, and log-likelihood − 11.9).

## Discussion

In this work, we studied the added value of texture features extracted from native T1 mapping on LGE assessment in DMD patients. Our findings suggest that one of four features, IDMN or inverse difference moment normalized, is a good option for such a purpose. In the study context, this feature measures the uniformity of the pixel intensity levels within the myocardium in native T1 maps [46]. It plays a crucial role in refining the spatial variations in pixel values derived from T1 parametric mapping. Therefore, it might help to detect slight alterations where regions with LGE typically exhibit distinct intensity patterns compared to healthy tissue, such as those reported in fibrosis or inflammation [5, 6], without using GBCA.

### Texture analysis

Native T1 mapping provides valuable information about myocardial tissue [5, 6], and our results suggest that including IDMN in the assessment might offer a distinct advantage. It enhances the sensitivity of T1 mapping in identifying myocardial alterations and is a non-invasive approach to detect early signs of disease progression. Furthermore, the IDMN feature used the wavelet low-low filter, which focuses on capturing the low-frequency components of the image, emphasizing the broader patterns and textures present in the cardiac MRI native T1 maps while reducing the influence of finer details and noise.

Texture analysis (TA) of native T1 mapping has been used to identify and classify hypertrophic cardiomyopathy [29, 32], diagnose acute myocarditis [28], study heart failure [34], and even characterize diffuse fibrosis patterns [59]. However, to our knowledge, this is the first study focusing on TA in DMD patients intending to impact the use of GBCA in their yearly clinical examinations. As cardiac MRI examinations already pose a challenge for some DMD patients, limiting the time they spend inside the scanner and minimizing GBCA use in this pediatric population would be optimal for detecting early myocardial involvement [3, 12, 20]. Moreover, TA has potential value for patients where administering contrast agents is not feasible, such as in cases with difficult venous access or identification, patient discomfort, anatomical positions that hinder catheter insertion, and potential vascular fragility or compromised circulation associated with DMD.

### Native T1 parametric mapping

It has been shown that DMD patients have higher native T1 relaxation times than healthy subjects, regardless of myocardial fibrosis assessed by LGE [43, 60–62]. However, there are contradictory reports regarding identifying LGE status from native T1 maps when comparing DMD patients with and without LGE [43, 60, 61, 63, 64]. Studies found similar values in the segmental and global native T1 relaxation times [43], higher values for LGE + DMD patients limited to the lateral wall [60, 61] or the global assessment [60, 63], and even lower values for LGE + DMD patients compared to those without LGE [64]. We also found higher but not significant global native T1 relaxation times between patients with and without LGE. LVEF was preserved in all DMD, regardless of LGE status, as shown in other studies [65].

These contradictory results regarding native T1 relaxation times might benefit from adding an extra layer of detail, considering also that a previous study showed that, by itself, native T1 parametric mapping is not a strong predictor of LGE in DMD [18]. From our findings, IDMN leverages the information in T1 parametric mapping, refining the spatial variations in the pixel values and identifying subtle alterations in tissue texture. As a result, it might complement the utility of native T1 mapping in identifying potential LGE in DMD patients without using GBCA. Furthermore, we found a significant effect on the logs of LGE by a preliminary assessment of this feature over time, suggesting that IDMN has a strong potential to help predict LGE progression in DMD patients, and monitoring this feature could also help reduce the frequency of GBCA use in their cardiac MRI examinations.

Finally, the clinical relevance of our study lies in the potential of TA to complement native T1 parametric mapping in DMD patients, considering the challenges they might experience during cardiac MRI examinations [20]. Even though only four of our participants had difficult peripheral venous access, this issue is commonly encountered in clinical practice [22]. Together with the lower proportion of DMD sufferers who might potentially have renal involvement [19, 21], it reinforces the need for alternative techniques. TA could initially be part of research assessments and, after further validation and automation, be incorporated gradually into existing routine clinical workflows [37]. Developing a pipeline adapted to specific clinical needs could provide clinicians with two main benefits: the ability to exploit native CMR examinations for particular patient subgroups when required and the flexibility to extend texture analysis models by integrating relevant clinical data, potentially enabling more advanced applications such as risk stratification tools to improve prognosis in DMD patients. Building on this approach, enhancing non-contrast imaging has the potential to optimize cardiac MRI examinations for DMD patients, improving their disease monitoring while minimizing GBCA use.

### Limitations

Our study has limitations. It was retrospectively performed in a single center, so our sample size was small. Additionally, our results were not externally validated, limiting their clinical applicability and generalizability. Nonetheless, the TA approach followed in this study can be extended to other pathologies beyond DMD. Even though more types of texture analysis exist in the literature, our study is based on one that has been consistently applied in cardiac MRI. Likewise, although we minimized patient selection bias, we could not rule it out completely, and we worked with artifact-free images. Even though they might not always represent the clinical practice, it is necessary to ensure the utility of texture features, as artifacts can significantly impact their assessment and compromise their reliability. Finally, even though there are conventional and widely studied clinical markers that predict LGE, we wanted to contribute to understanding how specific features can independently provide insight into LGE in DMD patients to advance knowledge in cardiac imaging. Future research will benefit from collaborative efforts to study the utility of IDMN assessment in DMD patients, establish a direct correlation between T1 mapping-derived TA features and their long-term clinical outcomes, and analyze the feasibility of reducing the frequency of GBCA use during their regular controls.

## Conclusion

We successfully implemented a pipeline for extracting texture features from cardiac MRI native T1 mapping and studied their potential in assessing LGE in DMD patients. Our findings showed that IDMN, a feature that measures the homogeneity of pixel values in a region, might aid in detecting slight myocardial tissue alterations associated with LGE, which is crucial for monitoring the cardiovascular health in these patients. Furthermore, IDMN might leverage the information native T1 parametric mapping provides, helping to reduce the use of GBCA in these patients’ yearly cardiac MRI examinations and aiding those with difficult venous access.

## Electronic supplementary material

Below is the link to the electronic supplementary material.

Supplementary Material 1

## Data Availability

The datasets used and/or analysed during the current study cannot be publicly shared because of patient’s privacy but are available from the corresponding author on reasonable request.

## Abbreviations

AIC: Akaike information criterion
AUC: Area under the curve
BIC: Bayesian information criterion
CI: Confidence interval
CMR: Cardiovascular magnetic resonance
CV: Coefficient of variation
DMD: Duchenne muscular dystrophy
EDV: End-diastolic volume
EF: Ejection fraction
ESV: End-systolic volume
FW: Free wall
GBCA: Gadolinium-based contrast agent
GLCM: Gray-level co-occurrence matrix
GLDM: Gray-level dependence matrix
GLM: Generalized linear mixed
GLRLM: Gray-level run length matrix
GLSZM: G ray-level size-zone matrix
ICC: Intraclass correlation coefficient
IDMN: Inverse difference moment normalized
ISP: Intelli Space Portal
LBP: Local binary pattern
LGE: Late gadolinium enhancement
LV: Left ventricle
LVM: Left ventricular mass
MAPSE: Mitral annular plane systolic excursion
MOLLI: Modified Look-Locker inversion recovery
NGTDM: Neighbouring gray-tone-difference matrix
NifTI: Neuroimaging Informatics Technology Initiative
RMS: Root mean squared
ROC: Receiver operating characteristic
RV: Right ventricle
SSFP: Steady-state free precession
SV: Stroke volume
TA: Texture analysis
TAPSE: Tricuspid annular plane systolic excursion
VIF: Variable inflation factor
WT: Wall thickness
