# Supplementary material for: Texture analysis of cardiovascular MRI native T1 mapping in patients with Duchenne muscular dystrophy
Source: Orphanet J Rare Dis. 2025 Mar 19;20:136. doi: 10.1186/s13023-025-03662-y (PMC11924673; doi:10.1186/s13023-025-03662-y)
Supplement: Supplementary file 1 — Supplementary Material 1 [file 13023_2025_3662_MOESM1_ESM.pdf]

Supplementary Table 1. Description of the features extracted with PyRadiomics according to the documentation [1].

Family	Description	Features
First order	Describe the distribution of voxel intensities within the image region defined by the mask through commonly used and basic metrics.	10 percentile, 90 percentile, energy, entropy, interquartile range, kurtosis, maximum, mean absolute deviation, mean, median, minimum, range, robust mean absolute deviation, root mean squared, skewness, total energy, uniformity, variance
Gray-level co-occurrence matrix (GLCM)	Describe the joint probability of finding specific combinations of pixel intensity pairs within a masked image region by analyzing the frequency of co-occurrences of gray levels separated by defined distances and angles.	autocorrelation, cluster prominence, cluster shade, cluster tendency, contrast, correlation, difference average, difference entropy, difference variance, ID (inverse difference), IDM (inverse difference moment), IDMN (inverse difference moment normalized), IDN (inverse difference normalized), IMC1 (informational measure of correlation 1), IMC2 (informational measure of correlation 2), inverse variance, joint average, joint energy, joint entropy, MCC (maximal correlation coefficient), maximum probability, sum average, sum entropy, sum squares
Gray-level dependence matrix (GLDM)	Quantify gray level dependencies in an image by counting the number of connected voxels within a defined distance that depends on the center voxel.	dependence entropy, dependence non-uniformity normalized, dependence non-uniformity, dependence variance, gray level non-uniformity, gray level variance, high gray level emphasis, large dependence emphasis, large dependence high gray level emphasis, large dependence low gray level emphasis, low ray level emphasis, small dependence emphasis, small dependence high gray level emphasis, small dependence low gray level emphasis
Gray-level run length matrix (GLRLM)	Quantify gray level runs (length in number of pixels) of consecutive pixels with the same gray value within a region of interest.	gray-level non-uniformity normalized, high gray level run emphasis, long run emphasis, long run high gray level emphasis, long run low gray level emphasis, low gray level run emphasis, run entropy, run length non-uniformity normalized, run length non-uniformity, run percentage, run variance, short-run emphasis, short-run high gray level emphasis, short-run low gray level emphasis
Gray-level size-zone matrix (GLSZM)	Quantify gray level zones, i.e., the number of connected voxels that share the same gray level intensity, in an image.	high-gray level zone emphasis, large area emphasis, large area high gray level emphasis, large area low gray level emphasis, low gray level zone emphasis, size zone non-uniformity normalized, size zone non-uniformity, small area emphasis, small area high gray level emphasis, small area low gray level emphasis, zone entropy, zone percentage, zone variance
Neighbouring gray-tone-difference matrix (NGTDM)	Quantify the difference between a gray value and the average gray value of its neighbors within distance in an image.	busyness, coarseness, complexity, strength
Shape	Features independent of the gray level intensity distribution in the ROI. They include the ROI's size and shape (two-dimensional) and are calculated using the non-derived image and mask.	elongation, major axis length, maximum diameter, mesh surface, minor axis length, perimeter surface ratio, perimeter, pixel surface, sphericity

Supplementary Figure 1. Boruta feature importance. Confirmed features are shown in red, tentative in blue, rejected in beige, and shadow features, artificially generated features within the algorithm for comparison, in green. The prefixes in the x-axis labels corresponding to the highly reproducible features start with the name of the family type in lowercase, i.e., firstorder (first-order features), glcm (gray-level co-occurrence matrix), gldm (gray-level dependence matrix), glrlm (gray-level run length matrix), glszm (gray-level size-zone matrix), ngtdm (neighbouring gray-tone-difference matrix), shape2D (shape features). Likewise, the suffixes in the x-axis labels are related to the type of treatment the image had, i.e., n (only normalization), gr (gradient filter), sq (square filter), lbp (local binary pattern; the radius 1 or 2 is indicated as r1 or r2), and w (wavelength; the sub-band filters are clarified by the subsequent lowercase letters, l for low, h for high).

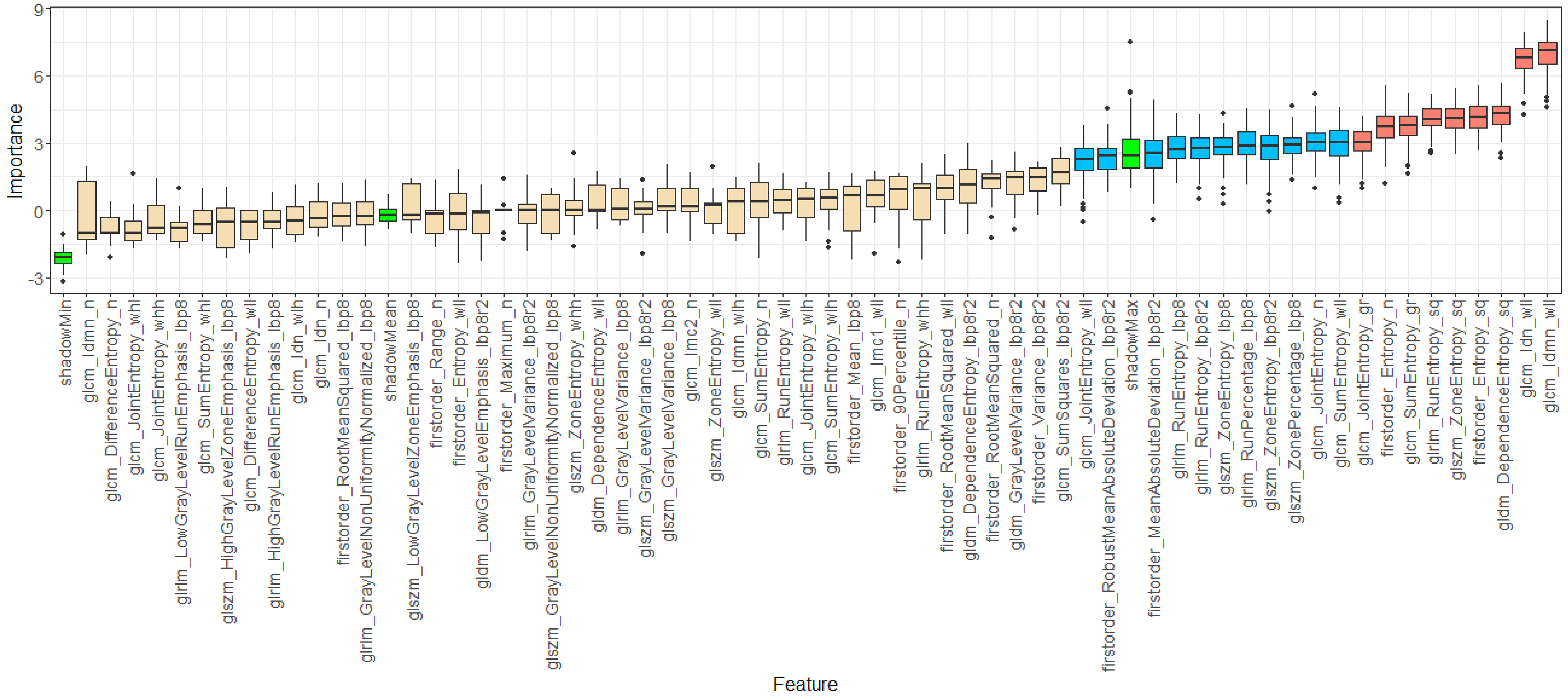

Supplementary Figure 2. LASSO relative feature importance. Features with positive coefficients are shown in blue, and those with negative coefficients are shown in red. The prefixes in the y-axis labels corresponding to the highly reproducible features start with the name of the family type in lowercase, i.e., firstorder (first-order features), glcm (gray-level co-occurrence matrix), gldm (gray-level dependence matrix), glrlm (gray-level run length matrix). The suffixes in the y-axis labels relate to the filter applied, i.e., n (only normalization), gr (gradient filter), sq (square filter), lbp (local binary pattern; the radius 1 or 2 is indicated as r1 or r2), and w (wavelet; the sub-band are clarified by the subsequent lowercase letters, l for low, h for high).

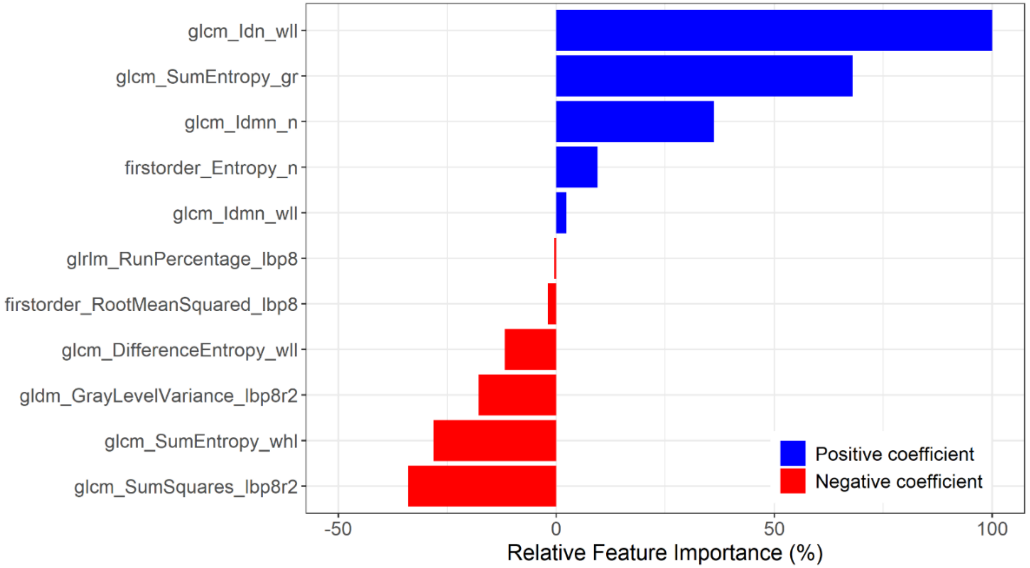

Supplementary Table 2. Variance Inflation Factor (VIF) of features selected by hierarchical clustering.

Variables	Type	Filter	VIF
Root mean squared	First order	LBP(8,1)	1.604
Entropy	First order	-	1.366
IDMN	GLCM	wavelength LL	3.200
Sum entropy	GLCM	gradient	2.085
Zone percentage	GLSZM	LBP(8,1)	2.303

Abbreviations: GLCM, gray-level co-occurrence matrix; GLDM, gray-level dependence matrix; GLSZM, gray-level size-zone matrix; IDMN, inverse difference moment normalized; LBP, local binary pattern; LL, low-low; VIF, Variance inflation factor.

References

1 van Griethuysen JJM, Fedorov A, Parmar C et al (2017) Computational Radiomics System to Decode the Radiographic Phenotype. Cancer Res 77:e104-e107
